# From coop to table: How increased welfare conditions shape chicken meat quality

**DOI:** 10.1016/j.psj.2025.105767

**Published:** 2025-09-01

**Authors:** Joanna Składanowska-Baryza, Ewa Sell-Kubiak, Patryk Sztandarski, Aneta Jaszczyk, Joanna Marchewka, Agnieszka Ludwiczak

**Affiliations:** a Poznań University of Life Sciences, Department of Animal Breeding and Product Quality Assessment, Słoneczna 1, 62-002 Poznań, Poland; b Poznań University of Life Sciences; c Department of Genetics and Animal Breeding, Wołyńska 33, 60-637 Poznań, Poland, Poznań University of Life Sciences; d Institute of Genetics and Animal Biotechnology of the Polish Academy of Sciences, Jastrzębiec, 05-552 Magdalenka, Poland

**Keywords:** Broiler chicken, Genotype, Environmental enrichment, Meat quality, Myopathy

## Abstract

This study aimed to evaluate the effects of two housing systems (free-range vs. indoor) and two slow-growing chicken genotypes (Hubbard JA 757 and JA 787) on breast meat quality traits relevant to consumer acceptance and processing. A total of 120 Hubbard broilers were divided into four groups based on genotype and housing conditions (30 birds per group). Physicochemical properties of the *Pectoralis major* muscle were analyzed post-slaughter (both fresh and frozen) to assess pH, color, water-holding capacity, texture, chemical composition, and incidence of muscle myopathies (Spaghetti Meat, Wooden Breast, and White Striping). Among the evaluated parameters, only the housing × genotype interaction significantly affected pH48. Housing system significantly influenced b* and C* color values (*p* = 0.0001), suggesting changes in meat pigmentation due to environmental conditions. JA757 birds exhibited higher thaw loss, while cooking loss was significantly affected by both genotype and housing, as well as their interaction. Texture analysis revealed that genotype significantly affected Young’s Modulus at both the 0–10 % and 20–80 % ranges, while housing conditions impacted the latter. Spaghetti Meat was more prevalent in JA787 birds and those raised indoors (*p* = 0.0001 and *p* = 0.01, respectively), whereas Wooden Breast and White Striping were not significantly influenced by genotype or housing. Fat content was significantly influenced by the housing system and its interaction with genotype, while protein and moisture contents remained stable across treatments. Genotype had a stronger influence on textural attributes and myopathy incidence, while housing conditions primarily shaped pH, pigmentation, water retention, and fat content. These findings underline the importance of aligning broiler genotype with rearing environment to optimize meat quality. Free-range systems, particularly in combination with the JA757 genotype, may better suit premium markets focused on animal welfare and tenderness, while indoor systems with JA787 birds may be better adapted to processed meat production.

## Introduction

Farming broilers with access to free range is becoming increasingly popular as it aligns with consumer preferences for ethically sourced, high-quality meat. Nevertheless, the specific effects of free-range systems on meat attributes—such as tenderness, color, flavor, and nutritional composition—remain under-investigated and a vital area of investigation. In conventional intensive systems, broilers are usually housed indoors on litter with high stocking densities (Castellini et al., 2002; Fanatico et al., 2005). Environmental factors, such as high stocking density and limited mobility, increase physiological and oxidative stress in broilers, as indicated by an elevated heterophil-to-lymphocyte ratio and reduced glutathione levels. These stress responses are associated with changes in meat characteristics, including a decrease in intramuscular fat content, which may affect texture and palatability. Thus, free-range systems offer animals more space so they have the opportunity to exhibit natural behavior, which can improve meat quality by alleviating stress and promoting optimal muscle development (Broom et al., 1991; Sørensen et al., 2000; Fanatico et al., 2007).

Numerous countries, including those in the European Union, have established regulations to improve livestock rearing conditions (Glatz and Pym, 2013; Council of the European Union, 2007). As consumers become more inclined to buy meat of a higher standard of production, poultry producers can adapt their products to meet market needs. Research into free-range broiler housing systems facilitates the establishment of standardized best practices, ensuring consistent product quality while complying with changing legislative mandates.

Also, free-range systems have been linked to ‘healthier’ meat, which has reduced fat content and a more favorable fatty acid profile, resonating with consumer preferences for nutritious food options . Despite the growing adoption of free-range farming, there are still significant knowledge gaps regarding its comprehensive impact on meat quality parameters While existing studies suggest that free-range poultry exhibits elevated levels of polyunsaturated fatty acids and superior sensory attributes, further empirical research is required to quantify these advantages across varying environmental conditions (Marchewka et al., 2023, Sztandarski et al., 2025). Furthermore, climatic variations, nutrition, and the response of individual genotypes to free-range housing require further research.

Beyond housing conditions, genetic predisposition plays a key role in determining both animal welfare and meat traits (Petracci et al., 2014). Selective breeding for traits such as accelerated growth rate and high feed conversion efficiency has had a huge impact on modern poultry production (Fanatico et al., 2007). However, these breeding strategies may violate welfare standards (vis-à-vis currently accepted standards) and, therefore, negatively affect meat texture, muscle fiber integrity, and fat deposition. Intensive genetic selection for rapid-growth broilers has been associated with muscle abnormalities and conditions such as pale, soft, and exudative (PSE) meat, which adversely affect consumer acceptance. In contrast, slow-growing heirloom breeds - such as Padovana and Polverara - showed better sensory properties, including improved tenderness and enriched flavor profiles (Pellattiero et al., 2020). If we are keen to obtain the highest quality raw material, in line with ethical farming practices, understanding genetic determinants is crucial to optimizing meat production.

This study investigates the effects of two housing systems (indoor with access to free-range and indoor) and two slow-growing chicken genotypes (Hubbard JA 757 and JA 787) on meat quality parameters critical in consumer purchasing decisions for raw poultry products. The findings aim to support more sustainable poultry production practices by combining high meat quality with ethical and environmentally responsible farming methods, thereby aligning with both consumer preferences and industry standards

## Materials and methods

### Animals and the farm environment

The experiment was conducted on the farm of the Institute of Genetics and Animal Biotechnology, Polish Academy of Sciences, located in the Mazovian region of Poland (52.032404, 20.826490), between July and September 2023.

The experimental procedures described did not require approval from the National Ethical Commission at the Ministry of Science and Higher Education in Poland, as they fell below the defined threshold of pain equivalent to a needle prick (Directive 2015/266/EC; Public Information Bulletin, 20203). Ethical Committee approval was not required for this study, as no invasive procedures or any interventions were conducted on the birds throughout their lifespan. Carcass evaluation followed EU standards (Regulation (EC) No. 543/2008). strictly adhering to routine husbandry activities included within typical broiler flock management protocols, following relevant national and EU legislation, as well as farming best practices and guidelines for each genotype.

The 120 Hubbard birds were divided into groups according to the genotype (JA 757 and JA 787) and housing system (Indoor and Free-range access) (Table 1). Each group contained five replications of the treatment group, with six birds in each replication, resulting in a total of 30 birds per group. Birds were kept in pens with sawdust bedding (0.96 *m* × 0.96 m and 0.92m² area each) at a stocking density of 21 kg/m² at slaughter age. During the first two days of the experiment, birds were maintained under continuous lighting (24 hours of light per day) to encourage early feed intake and facilitate adaptation to the environment. From day 3 onward, birds were exposed to a photoperiod consisting of 15 hours of natural light and 9 hours of darkness per day. Natural illumination was provided through uncovered windows, with a window-to-floor area ratio of 1:7. Each pen was equipped with a temperature and humidity monitoring system. The ambient temperature was initially set at 34 °C on day 0 and was gradually reduced to 19–21 °C by the end of the rearing period, while indoor relative humidity was controlled at approximately 45 %, a lower-bound setpoint used to limit litter moisture and ammonia under the prevailing summer conditions.

**Table 1:** Experimental design.

	INDOOR (*N* = 60)	FREE-RANGE (*N* = 60)
JA757 (*N* = 60)	(*N* = 30)	(*N* = 30)
JA787 (*N* = 60)	(*N* = 30)	(*N* = 30)

All Hubbard chickens were fed a certified organic diet, formulated to meet the nutritional needs of medium-growing broilers. Feed was provided in pelleted form and offered ad libitum alongside unrestricted access to fresh water. The feeding program included four nutritional phases: starter (0–10 days), grower 1 (11–23 days), grower 2 (24–48 days), and finisher (>48 days). The diet provided metabolizable energy (ME) levels of 3155–3240 kcal/kg and crude protein (CP) ranging from 21.3 % in the starter to 18.6 % in the finisher phase. Crude fat increased from 5.0 % to 5.5 %, crude fiber declined from 3.8 % to 2.6 %, lysine content decreased from 1.34 % to 1.10 %, and methionine ranged from 0.45 % to 0.50 % across the phases. Diet ingredients included corn, wheat, soybean meal, sunflower meal, dehulled sunflower seeds, potato processing by-products, poultry-derived animal fat, crude sunflower oil, and wheat bran, along with organic-approved vitamin–mineral premixes and zootechnical additives (e.g., phytase, xylanase, mannanase).

Indoor birds were kept in indoor pens with no outdoor access. Free-range birds were kept in indoor pens with access to the fenced pasture through a pop-hole (45 × 50 cm) opened daily from 7:00 to 19:00. Each genotype group (JA 757 and JA 787) had access to its own pasture (10 m x 3 m, 30m2). Pastures were characterized by a homogeneous botanical composition, with the plant cover cut a week before the start of the experiment and had no shelter. Each pasture was equipped with a semi-automatic bell drinker.

At 56 days the birds were slaughtered by a cervical dislocation and bleeding out. The slaughter was conducted by trained personnel. The average body weight for Hubbard hybrids was approximately 3030 g for the JA757, with a standard variation of about 60 g, while the JA787 chickens averaged 3025 g, with a variation of about 55 g.

After cooling the carcasses for 12 hours in +4°C, the *Pectoralis major* muscles (left side - A and right side - B) and legs were dissected from each carcass. The carcass weight, weight of both breast muscles, and both legs were recorded, and used to calculate the dressing out percentage, and the share of breasts and legs in the carcass. The muscles were individually sealed in plastic containers and transported under cold storage conditions (2–5°C) to the laboratory for comprehensive evaluation of meat quality.

### Meat quality examination

Each *Pectoralis major* muscle was cut into upper, middle, and lower parts. The upper part was subjected to pH measurement, weighed, packed in a low vacuum, and frozen (−20°C) for thaw loss, pH, cooking loss, and texture measurement. The middle parts were used for color and EZ drip measurements, whereas the lower parts were used for chemical composition examination. The pH was measured using a glass-calomel electrode (Lo 406- M6-DXK-S7/25, Mettler Toledo, Columbus, OH, USA) connected to a temperature-compensated pH meter (type 1140, Mettler Toledo, Columbus, OH, USA). The color was measured using a CM-700d spectrophotometer (Konica Minolta, Amsterdam, The Netherlands). The methodologies used for pH, color, thaw loss, Warner–Bratzler texture measurement, and basic chemical composition examination were described by Ludwiczak et al. (2024). The other methods used are described below.

The macroscopic scoring scheme of breast myopathies was performed 24 h after slaughter by three trained panellists. White Striping (WS), Wooden Breast (WB), and Spaghetti Meat (SM) were evaluated using established macroscopic scoring systems:

The incidences of myopathies were examined as nominal traits:

1. Spaghetti meat (0-absent; 1-present);
2. Woody breast (0-absent; 1-moderate; 2-severe);
3. White striping (0- absent; 1-mild; 2-moderate; 3-severe).

Drip loss was measured using the EZ-DripLoss method (Oksama, 2004). Briefly, two 2.0-cm steaks were sampled from each breast 48 h post-mortem, placed in EZ-DripLosscontainers, and stored at 4°C for 24 h. Drip loss was expressed as percentage weight loss

The calculation of EZ-Driploss was conducted according to the following formula:

EZ−Driploss=[(W1−Wc)/(Wt−−Wc)]×100

Where:

Wc - is the weight of the empty EZ-DripLoss container;
Wt - is the weight of the EZ-DripLoss container with meat and juice;
W1 - is the weight of the EZ-DripLoss container with juice.

The cooking loss of the thawed samples was measured over two months at –20°C, using the modified method of Honikel (1998). The upper parts of the pectoralis major muscles were weighed and packed in polyethylene bags meant for the thermal processing of food (sous vide bags; 67 g/m^2^;; density; oxygen permeability; ≤ 4.0 g/m2; ≤ 65 cm3/m 2′d*bar/24 h water vapour permeability), with the bag’s wall firmly adhering to the meat sample. A calibrated thermocouple probe was inserted into the center of the additional sample. The samples were placed in a water bath preheated to 76°C and cooked until they reached an internal temperature of 72°C. The samples were removed from the water bath, immediately submerged in an ice bath, and reweighed (without a vacuum bag). Two methods of cooking loss measurement were applied.

Cooking loss – Samples were removed from the vacuum bags and placed in the refrigerator at 4°C. Then, the samples were reweighed after 12 hours. The weight loss was calculated via the following formula:

Cooking loss (%) = (weight loss after cooking *100)/weight before cooking

Meat texture was analyzed using the Blunt Meullenet–Owens Razor Shear (BMORS) test, performed on a TA.XT Plus Texture Analyzer (Stable MicroSystems, Warrington, UK). The BMORS method employs a blunt blade (24 mm height, 8 mm width, 0.5 mm thickness), which is designed to better simulate the mechanical action of chewing compared to sharp-blade shear tests. Each cooked breast sample was subjected to six shears perpendicular to the muscle fibers. The test parameters included: 20 mm penetration depth, crosshead speed of 10 mm/s, and a trigger force of 10 g. The following texture parameters were recorded: shear force (N), shear energy (N·mm), and Young’s Modulus (YM) at 0–10 % and 20–80 % compression. These measurements provided an objective assessment of the meat’s tenderness and structural properties.

Bone quality traits were assessed using a TA.XT Plus Texture Analyser (Stable MicroSystems, Warrington, UK) following the methodology of Shim et al. (2012). In addition, femur morphometrics were recorded: femur length (mm) was measured with a digital caliper from the femoral head to the distal condyles, and femur circumference (mm) was measured at the mid-shaft using a flexible tape. All measurements were taken in triplicate and averaged to ensure precision.

### Statistical analysis

All the statistical analysis were conducted in the R statistical package (version 4.3.2, R Foundation for Statistical Computing, Vienna, Austria). Data were first tested for normality using the Shapiro–Wilk test, and the Levene test was applied to verify homogeneity of variance. Differences between groups were analyzed using a two-way ANOVA with interaction, including the main effects of housing system (indoor vs. free-range), genotype (JA757 and JA787), and their interaction. When significant effects were detected, Tukey’s post hoc test was used for pairwise comparisons. Statistical significance was set at *P* < 0.05, and tendencies were discussed at 0.05 ≤ *P* < 0.10.

The incidence of myopathies (Wooden Breast, White Striping, Spaghetti Meat) was treated as a categorical variable and the effects of genotype and housing on the number of cases were compared between groups with the Chi-square test at *P* < 0.05.

## Results

The results of this study evaluated the effects of genotype (JA757 and JA787), housing system (free-range and indoor), and their interaction on various slaughter traits and parameters of broiler breast meat.

No significant differences were observed between genotypes or housing systems for slaughter weight, carcass weight, breast muscles, and legs weight (Table 2). Similarly, no significant interaction effects were found for these parameters. Dressing out percentage was significantly affected by housing conditions (*P* = 0.024), with indoor-reared birds showing slightly higher values compared to those reared in free-range systems, while no genotype or interaction effects were observed. The pH measured 24 hours post-slaughter (pH_24_) showed no significant differences across genotypes, housing systems, or interaction effects. At 48 hours, both the housing system (*P* = 0.040) and the housing × genotype interaction (*P* = 0.02) significantly affected pH_48_ values.

**Table 2:** Effect of genotype and housing type on carcass traits (on fresh chicken and legs) and physicochemical properties of fresh chicken breast.

Parameter	Genotype		*p*-value genotype	Housing conditions			*p*-value housing	*p*-value Interaction	SEM
	JA757	JA787		FREE-RANGE		INDOOR			
Slaughter weight (g)	3002.5	3043.1	ns	3063.4		2982.9	ns	ns	74.0
Carcass weight (g)	2248.6	2264.8	ns	2271.0		2242.7	ns	ns	60.7
Dressing out %	74.4	74.7	ns	74.0		75.1	0.024	ns	0.32
Breast muscles (g)	676.8	687.1	ns	681.6		682.9	ns	ns	25.0
Breast muscles %	30.3	30.1	ns	29.9		30.4	ns	ns	0.58
Legs (g)	357.3	351.0	ns	357.9		350.0	ns	ns	35.5
Legs %	15.6	15.9	ns	15.7		15.8	ns	ns	1.54
Breast muscle quality traits									
pH_24_	5.88	5.88	ns	5.85		5.91	ns	ns	0.72
pH_48_	5.82	5.85	ns	5.81		5.86	ns	0.040	0.02
Drip loss (%)	0.54	0.64	ns	0.66		0.51	ns	ns	0.07
L*	51.5	51.6	ns	51.7		51.4	ns	ns	0.42
a*	−0.04	−0.18	ns	−0.14		−0.07	ns	ns	0.09
b*	5.6	5.7	ns	6.1		5.3	0.0001	ns	0.17
C*	5.6	5.8	ns	6.1		5.3	0.0001	ns	0.17
h*	90.4	91.8	ns	91.6		90.5	ns	ns	1.02

Regarding EZdrip loss (EZ%), no significant differences were noted for genotype, housing conditions, or interaction between these effects. For the meat color parameters, L* (brightness), a* (redness), C* (Chroma), and *h* (hue) showed no significant effects across genotype, or interaction. Indoor-housed birds had significantly lower b* and C* values compared to free-range birds (*P* = 0.0001), whereas L*, a*, and h* values were not influenced by the housing system (Table 2).

According to Table 3, the analysis of thawed broiler breast meat revealed significant influences of genotype, housing system, and their interaction on several quality parameters. For pH value, a substantial interaction between fixed effects was found.

**Table 3:** Effect of genotype and housing conditions. on the physicochemical qualities and texture of thaw chicken breast.

Item	Genotype		*p*-value Genotypes	Housing conditions		*p*-value Housing	*p*-value Interaction	SEM
	JA757	JA787		FREE-RANGE	INDOOR			
pH	5.87	5.83	ns	5.86	5.83	ns	0.039	0.13
Thaw loss %	11.6	8.1	0.001	10.5	9.3	ns	0.007	0.6
Cooking loss %	11.1	8.2	0.001	10.4	9.0	0.054	0.001	0.7
Warner Bratzler								
Force N	17.6	16.4	ns	16.6	17.4	ns	ns	2.3
Energy N*mm	157.1	140.9	ns	136.7	161.1	0.021	ns	5.5
YM 0-10 %	0.29	0.24	0.002	0.26	0.27	ns	0.053	0.03
YM 20-80 %	2.49	1.98	0.001	2.07	2.40	0.043	ns	0.19
BMORS								
Force N	9.6	8.9	ns	9.8	8.7	ns	ns	1.6
Energy N*mm	126.9	96.2	0.001	108.8	114.3	ns	ns	4.1
YM 0-10 %	0.53	0.37	0.001	0.39	0.51	0.001	0.001	0.03
YM 20-80 %	0.71	0.75	ns	0.83	0.63	ns	ns	0.07

YM – Young Modulus.

Cooking loss was significantly greater in JA757 compared to JA787 (*P* = 0.001). A tendency for higher values was also observed in free-range compared to indoor birds (*P* = 0.054). Importantly, a significant housing × genotype interaction was found (*P* = 0.001).

Thaw loss showed significant differences between genotypes, with JA757 meat exhibiting higher loss than JA787 and a notable interaction effect, although housing alone did not have a considerable impact.

Warner-Bratzler measurements revealed significant genotype effects for 0-10 % YM and 20-80 % YM, with the housing system also influencing the results for the 20-80 % YM. While Warner-Bratzler energy was significantly affected by the housing system, no genotype or interaction effects were observed for Warner-Bratzler force or energy. The BMORS test results indicated that genotype significantly influenced energy and 0-10 % YM, with the housing system also affecting 0-10 % YM. BMORS 0–10 % Young’s Modulus was significantly influenced by genotype (*P* = 0.001), housing system (*P* = 0.001), and their interaction (*P* = 0.001).

A total of 61 broilers were examined for the presence of three major breast muscle myopathies: Spaghetti Meat (SM), Wooden Breast (WB), and White Striping (WS), with comparisons made across two genotypes (JA757 and JA787) and two housing systems (free-range and indoor) (Table 4). The incidence of Spaghetti Meat was slightly higher in JA757 (28 out of 32 birds) compared to JA787 (23 out of 29 birds), though this difference was not statistically significant (*P* = 0.388). Similarly, housing system did not significantly influence the occurrence of SM, with 24 of 31 free-range birds and 27 of 30 indoor birds showing signs of the condition (*P* = 0.184).

**Table 4:** The effect of genotype and housing on the incidence of myopathies in *Pectoralis major.*

		Genotype			Housing		
		JA757 (*n* = 32)	JA787 (*n* = 29)	*p*-value	FREE-RANGE (*n* = 31)	INDOOR (*n* = 30)	*p*-value
SM	No (*n* = 10)	4	6	0.388	7	3	0.184
	Yes (*n* = 51)	28	23		24	27	
WB	No (*n* = 34)	15	19	0.532	17	17	0.837
	Yes: 1 (*n* = 16)	10	6		8	8	
	Yes: 2 (*n* = 6)	4	2		4	2	
	Yes: 3 (*n* = 5)	3	2		2	3	
WS	No (*n* = 46)	27	19	0.076	25	21	0.268
	Yes: 1 (*n* = 10)	2	8		3	7	
	Yes: 2 (*n* = 5)	3	2		3	2	

SM – Spaghetti meat; WB – Wooden breast; WS – White striping.

Wooden Breast was also distributed similarly across genotypes, with 15 cases found in JA757 and 19 in JA787, showing no statistically significant difference (*P* = 0.532). The severity grades of WB (mild to severe) were evenly distributed across both genetic lines. Housing condition had no observable effect on the prevalence of WB either, with both free-range and indoor groups each having 17 birds free of WB (*P* = 0.837), and a comparable distribution across severity levels.

Although not statistically significant, the difference in White Striping prevalence between JA787 (10 cases) and JA757 (5 cases) showed a trend (*P* = 0.076), warranting further investigation. The housing system again did not significantly affect the incidence of WS, with 6 cases among free-range birds and 9 among those kept indoors (*P* = 0.268).

Fat content was significantly affected by the housing system (*P* = 0.03) and the housing × genotype interaction (*P* = 0.05). A tendency for genotype effects was also noted (*P* = 0.07). However, the evaluated factors had a largely unaffected moisture and protein content. These results highlight the potential for targeted environmental and genetic interventions to optimize specific compositional traits of broiler breast meat (Table 5).

**Table 5:** Effect of Genotype and Housing Conditions on the Proximate Composition (%) of Chicken Breast.

Item	Genotype		*p*-value Genotype	Housing system		*p*-value Housing	*p*-value Interaction	SEM
	JA757	JA787		FREE-RANGE	INDOOR			
Moisture %	73.64	75.38	0.350	74.36	74.86	ns	ns	1.08
Fat %	1.11	4.10	0.070	0.47	4.92	0.030	0.050	1.14
Protein %	24.22	23.36	0.100	23.87	23.65	ns	ns	0.39

The analysis of leg parameters demonstrated that genotype significantly influenced pH (*P* = 0.009) and Warner-Bratzler energy (*P* = 0.009), with JA757 exhibiting higher pH and JA787 showing higher Warner-Bratzler energy (Table 6). A significant interaction effect was observed for pH (*P* = 0.036), indicating that the combination of genotype and housing system influenced this parameter. However, no significant differences were found for the housing system alone in terms of pH or Warner-Bratzler energy.

**Table 6:** Effect of Genotype and Housing Condition on Bone and Meat Quality Traits in Chicken Legs.

Parameter	Genotype		*p*-value Genotype	Housing system		*p*-value Housing	*p*-value Interaction	SEM
	JA757	JA787		FREE-RANGE	INDOOR			
Femur length (mm)	8.60	9.20	0.240	9.08	8.8143	ns	ns	0.30
Femur circumference (mm)	9.54	10.67	0.240	10.47	9.9314	ns	ns	0.50
MEAT pH	6.56	6.44	0.009	6.47	6.54	ns	0.01	0.03
MEAT Cooking loss %	13.35	15.17	0.430	16.11	12.41	ns	ns	1.59
MEAT Moisture %	74.28	73.52	0.170	73.81	73.98	ns	ns	0.36
MEAT Fat %	3.78	3.94	0.770	3.76	3.96	ns	ns	0.34
BONE Hardness N	121.0	112.1	0.600	117.1	116.0	ns	ns	11.0
BONE Brittleness/Flexibility mm	4.08	4.46	0.170	4.21	4.33	ns	ns	0.18
BONE Toughness N/mm	29.42	25.21	0.190	27.57	27.06	ns	ns	2.13
MEAT WB Firmness N	9.64	13.71	0.920	12.59	10.76	ns	ns	0.77
MEAT WB ENERGY N*mm	51.33	85.56	0.009	74.15	62.73	ns	ns	9.24
WB Young 0-10 %	0.20	0.25	0.080	0.23	0.22	ns	ns	0.02
WB Young 0-20 %	1.21	1.48	0.950	1.41	1.28	ns	ns	0.11

Other parameters, including femur length, bone circumference, cooking loss, moisture content, fat content, and bone properties (hardness, brittleness, and toughness), showed no significant influence of genotype, housing system, or their interaction. Similarly, Warner-Bratzler firmness and YM measurements (0-10 % and 0-20 %) were not significantly affected by any of the evaluated factors.

## Discussion

The results of the present study indicate that the genotype of broilers (JA757 and JA787) and housing conditions (FREE-RANGE and INDOOR) had a limited effect on fundamental carcass parameters, such as slaughter weight, carcass weight, and breast and thigh muscle weight. Free range was found to have an effect on the dressing out percentage only. The lack of significant variation in the majority of carcass parameters aligns with Fanatico et al. (2007) 's findings, which also observed that neither genotype nor housing system impacted broiler body weight at slaughter. This uniformity may be attributed to the consistent performance of modern broiler lines, which exhibit rapid weight gain regardless of environmental conditions (Petracci et al., 2019). Our findings suggest that while housing systems influence specific meat quality parameters (pH, color, and fat content), they do not significantly impact carcass composition in modern broilers. This may be attributed to genetic adaptability, sound management practices, and relatively uniform activity levels across housing conditions. Rather than contradicting Özbek et al. (2020), our results refine their conclusions, highlighting that the effects of housing on carcass traits are contingent on broiler strain, environmental management, and the duration of rearing.

Although the pH measured 24 hours post-slaughter (pH_24_) did not significantly differ between groups, the effect of housing conditions was observed after 48 hours (pH_48_; *P* = 0.04). Previous studies suggest that meat pH is influenced by locomotor activity, as free-range birds typically exhibit higher activity levels, which can affect muscle glycogen metabolism and result in a lower pH at 48 hours (Berri et al., 2005). Research conducted by Husak et al. (2008) compared meat quality parameters among organic, free-range, and conventionally raised broilers. They found that breast meat from organic broilers exhibited higher ultimate pH values than that from free-range and conventional broilers. This suggests that housing systems can influence the meat pH, potentially due to differences in pre-slaughter stress and muscle energy metabolism. However, contrary to these findings, Özbek et al. (2020) found that breast meat pH was significantly affected by both genotype and housing system at earlier post-slaughter intervals (15 min, 1 h, and 24 h). Such differences may result from variations in experimental design, including factors such as genotype, diet composition, pre-slaughter management, slaughter procedures, and post-slaughter carcass management and locomotor activity.

The present study revealed that housing conditions influenced meat color parameters, particularly the b* value (yellowness) and C* (colour saturation) (*p* = 0.0001). The C* parameter indicates overall color saturation, reflecting the vividness of the meat color, and is not limited to yellowness alone. Similar observations were made by Mudalal et al. (2014), who noted more intense meat coloration in broilers raised under intensive systems. This may be linked to reduced activity levels and higher intramuscular fat deposition. On the other hand, Fanatico et al. (2007) found that free-range broiler meat tends to be darker due to a higher myoglobin content, which was not confirmed in our study. Özbek et al. (2020) reported higher redness values (a*) in slow-growing birds and those reared on deep litter, attributed to increased oxidative muscle fiber activity. The combined findings suggest that producers seeking higher redness (a* values) in broiler meat might benefit from using slow-growing genotypes and rearing systems that promote moderate and consistent movement, such as deep litter systems.

Thaw loss was significantly influenced by genotype, with JA757 showing higher losses compared to JA787. This finding was similar to those obtained by Petracci et al. (2014), who demonstrated that genotype impacts the water-holding capacity of meat, potentially due to differences in muscle fiber structure. In the present work, water loss during cooking was significantly affected by genotype, housing system, and their interaction. Similarly, Pavlovski et al. (2009) highlighted that the water-holding capacity of broiler breast meat was lower in both semi-intensive and free-range systems. This finding suggests that housing systems can significantly impact meat quality parameters, possibly due to variations in environmental conditions and chicken activity levels.

Texture measurements using the Warner-Bratzler method showed that genotype significantly affected both Young’s Modulus (0–10 % and 20–80 %), while the housing system influenced the 20–80 % YM. These results are consistent with those of Kokoszyński et al. (2022), who compared Ross 308 and Cobb 500 broiler chickens and found significant differences in meat texture attributes, including hardness, springiness, chewiness, and gumminess. Specifically, Ross 308 chickens exhibited higher hardness, chewiness, and gumminess, while Cobb 500 chickens had greater springiness. Our results are also consistent with previous findings on slow-growing broiler genotypes, which generally exhibit improved meat quality traits under alternative housing systems (Castellini et al., 2008; Fanatico et al., 2007; Mikulski et al., 2011).

These variations were attributed to differences in muscle fiber characteristics between the genotypes. However, additionally to the genotype effect, the housing system may also contribute to a variation in meat texture by influencing biomechanical meat properties through differences in locomotor activity and muscle fiber density.

Our findings confirmed that Spaghetti Meat (SM) was significantly affected by both genotype and housing system, with a notably higher incidence observed in JA787 birds and those reared indoors. This aligns with the hypothesis proposed by Kuttappan et al. (2013), who indicated that reduced activity in intensive systems may exacerbate the development of muscular abnormalities due to insufficient muscle stimulation. In contrast, Wooden Breast (WB) and White Striping (WS) did not differ significantly across the experimental groups (*p* > 0.05), which suggests that these myopathies may not be directly linked to genotype or rearing conditions in slower-growing broilers. This is consistent with the observations of , who highlighted that such myopathies are primarily associated with rapid growth rates and metabolic stress rather than environmental or genetic variables. Although the prevalence of WS was numerically higher in JA787 compared to JA757 (10 vs. 5 cases), the trend (*P* = 0.076) did not reach statistical significance, indicating a possible weak genotype association that warrants further investigation. Collectively, these results support the notion that slow-growing genotypes, when managed under enhanced welfare conditions, exhibit reduced susceptibility to severe muscle disorders, particularly when housed in systems allowing for greater mobility.

Housing conditions significantly influenced fat content and interacted with genotype, indicating that the chemical composition of meat can be modulated through environmental adjustments. Funaro et al. (2014) found that broilers raised under intensive systems had a greater propensity for fat deposition. However, no significant differences were observed in protein or moisture content, which is consistent with the findings of Petracci and Berri (2017).

**Fig. 1:**
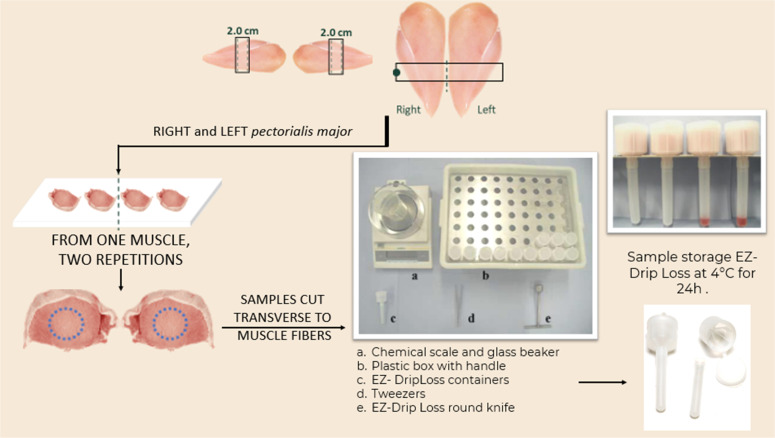
EZ-DripLoss procedure.

For leg meat properties, genotype significantly affected pH (*P* = 0.009) and Warner-Bratzler energy (*P* = 0.009). However, the studied factors did not substantially influence bone length, girth, and mechanical strength. Paxton et al. (2014), similarly to our research, found that overall bird health and activity levels largely determine the biomechanical properties of broiler bones. They found that while broilers exhibited rapid body mass gain, hindlimb bone length remained consistent during specific growth periods, which may contribute to increased stability. This suggests that factors beyond bone dimensions, such as overall health and activity levels, play a crucial role in maintaining bone integrity. Furthermore, significant genotype × housing system interactions were noted for leg pH (*P* = 0.015), corroborating the findings of Özbek et al. (2020). These results suggest that the relationship between housing conditions and meat quality traits varies by genotype, with muscle fiber structure dependent on differences in locomotor activity (Fig. 1).

The results of this study highlight the interactions between genotype and housing conditions in shaping meat quality traits. Housing systems affect meat pH, color parameters and fat content. These results suggest that producers could improve meat quality by tailoring the housing environment to specific chicken broiler genotypes. Future research should focus on refining breeding strategies and optimizing housing systems to enhance meat quality while maintaining high production efficiency.

In practical terms, the results demonstrate that while genotype had limited influence on carcass yield traits, it significantly affected meat texture characteristics and the occurrence of certain myopathies such as Spaghetti Meat. Concurrently, housing systems influenced meat pH measured at 48 hours post-slaughter, meat color parameters (b* and C*), water-holding capacity during thawing and cooking, and fat content. These findings enable producers to design targeted dual-market poultry production strategies based on market requirements and production goals.

For the premium meat market, where ethical rearing, tenderness, and nutritional quality are key consumer concerns, combining the JA757 genotype with free-range systems is advisable. This setup results in lower water and cooking loss, reduced Spaghetti Meat incidence, and more favorable fat levels—characteristics aligned with health-conscious and welfare-focused consumer preferences.

In contrast, for meat destined for the processing industry, where slightly firmer textures and higher fat content are acceptable or even beneficial for emulsified or structured products, the JA787 genotype raised under indoor conditions offers an efficient alternative. These birds exhibited higher shear force and firmness values, which can be advantageous for products such as sausages, marinated fillets, or restructured meats that benefit from a denser texture.

## Conclusion

In conclusion, the interaction between genotype and housing system plays a crucial role in determining various aspects of chicken meat quality, extending beyond basic carcass yield. Genotype primarily influences texture and susceptibility to myopathies, while environmental conditions affect color, pH, fat content, and water retention. These insights can be used to guide rearing practices that are both market-oriented and welfare-conscious, ultimately contributing to a more sustainable and economically viable poultry sector.

## Ethics approval

According to the Journal of Laws of the Republic of Poland, Item 266, Article 1 of the Act of 15 January 2015 on the Protection of Animals Used for Scientific and Educational Purposes in Poland, the study does not require the ethical approval of the Polish Ethical Commission. This experiment imitated standard on-farm production cycles, strictly adhering to routine husbandry practices without introducing any interventions beyond typical management protocols.

## Financial support statement

This study was conducted within the project entitled: “Linking extensive husbandry practices to the intrinsic quality of pork and broiler meat” - mEATquality, funded by the European Union’s Horizon 2020 Research and Innovation program under Grant Agreement No 354 101000344.

## CRediT authorship contribution statement

**Joanna Składanowska-Baryza:** Writing – review & editing, Writing – original draft, Validation, Data curation, Conceptualization. **Ewa Sell-Kubiak:** Writing – original draft, Resources, Project administration, Investigation, Funding acquisition, Formal analysis. **Patryk Sztandarski:** Writing – original draft, Resources, Methodology, Data curation, Conceptualization. **Aneta Jaszczyk:** Writing – original draft, Resources, Methodology, Investigation, Data curation, Conceptualization. **Joanna Marchewka:** Writing – original draft, Resources, Project administration, Methodology, Funding acquisition, Formal analysis, Data curation, Conceptualization. **Agnieszka Ludwiczak:** Software, Methodology, Investigation, Formal analysis, Data curation, Conceptualization.

## Disclosures

The Authors declare no conflict of interest.

## Appendix Supplementary materials

Image, application 1
