# Supplementary material for: From coop to table: How increased welfare conditions shape chicken meat quality
Source: Poult Sci. 2025 Sep 1;104(11):105767. doi: 10.1016/j.psj.2025.105767 (PMC12489921; doi:10.1016/j.psj.2025.105767)
Supplement: Supplementary file 1 [file mmc1.docx]

**FROM COOP TO TABLE: HOW INCREASED WELFARE CONDITIONS SHAPE CHICKEN MEAT QUALITY**

Joanna Składanowska-Baryza *, Ewa Sell-Kubiak †^2^, Patryk Sztandarski^‡^ , Aneta Jaszczyk^‡^, Joanna Marchewka^‡^, Agnieszka Ludwiczak *^1^

*Poznań University of Life Sciences, Department of Animal Breeding and Product Quality Assessment, Słoneczna 1, 62-002 Poznań, Poland, ^2^ Poznań University of Life Sciences,

† Department of Genetics and Animal Breeding, Wołyńska 33, 60-637 Poznań, Poland, Poznań University of Life Sciences,

^‡^ Institute of Genetics and Animal Biotechnology of the Polish Academy of Sciences, Jastrzębiec, 05-552 Magdalenka, Poland

**Table 7**

Feed composition and feeding schedule.

|  |  | Starter | Grower 1 | Grower 2 | Finisher |
| --- | --- | --- | --- | --- | --- |
| Feeding schedule (days) | | 0-9 | 10-20 | 21-32 | >32 |
|  | Energy (kcal/kg) | 3155.00 | 3100.00 | 3145.00 | 3240.00 |
|  |  |  |  |  |  |
| Feed analytical ingredients | Crude protein % | 21.30 | 19.90 | 18.7 | 18.6 |
|  | Raw ash % | 5.50 | 4.80 | 4.20 | 4.00 |
|  | Raw fat % | 5.00 | 5.10 | 5.20 | 5.50 |
|  | Raw fiber % | 3.80 | 3.40 | 2.70 | 2.60 |
|  | Lysine % | 1.34 | 1.21 | 1.12 | 1.10 |
|  | Calcium % | 0.80 | 0.6 | 0.50 | 0.50 |
|  | Phosphorus % | 0.52 | 0.45 | 0.37 | 0.34 |
|  | Methionine % | 0.45 | 0.53 | 0.51 | 0.50 |
|  | Sodium % | 0.15 | 0.15 | 0.15 | 0.15 |
|  |  |  |  |  |  |
| Feed supplements | Vitamin D/ 25-hydroxycholecalciferol (IU/kg) | 1000.00 | 1000.00 | 0.00 | 0.00 |
|  | Vitamin D3 (IU/kg) | 3000.00 | 3000.00 | 3000.00 | 3000.00 |
|  | Vitamin A (IU/kg) | 13000.00 | 10000.00 | 10000.00 | 10000.00 |
|  | Vitamin E (All-rac-alpha-tocopheryl acetate) (mg/kg) | 80.00 | 60.00 | 30.00 | 30.00 |
|  | Iron-Fe (Ferrous sulfate, monohydrate) (mg/kg) | 20.00 | 20.00 | 20.00 | 20.00 |
|  | Coated, granulated anhydrous calcium iodate, Iodine (mg/kg) | 1.00 | 1.00 | 1.00 | 1.00 |
|  | Copper-Cu (Copper sulfate pentahydrate) (mg/kg) | 8.00 | 8.00 | 8.00 | 8.00 |
|  | Copper-Cu (Copper trihydroxychloride) (mg/kg) | 7.00 | 7.00 | 7.00 | 7.00 |
|  | Manganese-Mn (Manganese oxide(II)) (mg/kg) | 80.00 | 80.00 | 80.00 | 80.00 |
|  | Zinc-Zn (Zinc sulfate, monohydrate) (mg/kg) | 40.00 | 40.00 | 40.00 | 40.00 |
|  | Zinc-Zn (Zinc hydroxychloride monohydrate) (mg/kg) | 35.00 | 35.00 | 35.00 | 35.00 |
|  | Sodium selenite, Selenium-Se (mg/kg) | 0.30 | 0.30 | 0.30 | 0.30 |
|  | Guanidinoacetic acid (mg/kg) | 0.00 | 573.00 | 573.00 | 573.00 |
|  |  |  |  |  |  |
|  |  |  |  |  |  |
| Feed zootechnical additives | Endo-1,4-beta-xylanase (U/kg) | 2428.00 | 2428.00 | 2428.00 | 2388.00 |
|  | Endo-1,3(4)-beta-glucanase (U/kg) | 302.00 | 302.00 | 302.00 | 0.00 |
|  | 6-phytase (FTU/kg) | 1493.00 | 1493.00 | 1990.00 | 1493.00 |
|  | Endo-1,4-beta-mannanase (U/kg) | 52537.00 | 52537.00 | 52537.00 | 52537.00 |
|  | *Bacillus lichiniformis* (CFU/kg) | 1.00 x 10^9 | 1.00 x 10^9 | 0.00 | 0.00 |

**Table 8**

Feed ingredients.

| Starter | Grower 1 | Grower 2 | Finisher |
| --- | --- | --- | --- |
|  |  |  |  |
| Corn, soybean meal, wheat, sunflower meal, sunflower seeds, calcium carbonate, oat,  rapeseed meal, animal fat (poultry), monocalcium phosphate,  vegetable oils and fats (sunflower-crude, sodium chloride, animal fat (pork), sodium sulfate  wheat bran, vegetable oils and fats (sunflower seeds) | Wheat, soybean meal,  sunflower seeds, dehulled sunflower seeds, triticale,  calcium carbonate, oat, wheat grain flour, calcium carbonate,  vegetable oils and fats (sunflower-crude)  monocalcium phosphate,  animal fat (poultry),  sodium chloride, sodium sulfate, wheat bran,  vegetable oils and fats (sunflower seeds),flax seeds | Corn, wheat, soybean meal, calcium carbonate, sunflower seeds, dehulled sunflower seeds, potato processing products, animal fat (poultry), vegetable oils and fats (sunflower-crude), sodium chloride, sodium sulfate, monocalcium phosphate, wheat bran, vegetable oils and fats (sunflower seeds) | corn, wheat, soybean meal, potato processing products, calcium carbonate, vegetable oils and fats (sunflower-crude), animal fat (poultry), sodium chloride, monocalcium phosphate, rapeseed meal, calcium carbonate, wheat bran, vegetable oils and fats (sunflower seeds) |

**Table 9**

Average Feed Intake of Ross 308 Broilers from day 1 to 41.

| Age (Days) | Phase | Feed Intake (g/bird/day) | Cumulative Feed Intake (g/bird) |
| --- | --- | --- | --- |
| 1-10 | Starter | 13-50 | 330-350 |
| 11-20 | Grower 1 | 55-100 | 900-1050 |
| 21-30 | Grower 2 | 110-150 | 1100-1300 |
| 31-41 | Finisher | 155-200 | 1300-1600 |
| Total (1-41) | Overall | - | 3630-4300 |
